# Acute thrombolysis in a patient with Type A aortic dissection and critical leg ischemia in an Arctic country

**DOI:** 10.1093/jscr/rjaf273

**Published:** 2025-05-03

**Authors:** Michala Norsell, Carsten Sauer-Mikkelsen, Sana Naseer Buttar, Luit Penninga

**Affiliations:** Ilulissat Hospital, Napparsimaviup Aqq. 4, Ilulissat 3952, Greenland; Ilulissat Hospital, Napparsimaviup Aqq. 4, Ilulissat 3952, Greenland; Clinic in Dermatology, Tolstrupvej 91, Stuen 1, 9700 Brønderslev, Denmark; Department of Cardiothoracic Surgery, Blegdamsvej 9, 2100 Copenhagen, Denmark; Ilulissat Hospital, Napparsimaviup Aqq. 4, Ilulissat 3952, Greenland; Department of Gastrointestinal Surgery, Aalborg University Hospital, Hobrovej 18-22 9000 Aalborg, Denmark

**Keywords:** Type A aortic dissection, remote hospitals, emergency medicine, thoracic surgery

## Abstract

A man in his early 50s presented at a remote hospital in Arctic Greenland with sudden severe chest and back pain, followed by critical ischemia in the right leg. Bedside ultrasound showed cessation of Doppler flow in the femoral artery, while ECG was normal. Aortic dissection was suspected, but lacking CT-scanner, MRI-scanner, or transesophageal ultrasound, the diagnosis could not be confirmed. The patient was treated symptomatically with thrombolysis for suspected femoral artery thrombosis. Overnight, the right leg regained color and pulses. The next day, weather circumstances allowed transfer to a secondary hospital, and CT-angiography showed a Type A aortic dissection extending from the aortic valve to both femoral arteries. Thrombolysis was stopped, and the patient was transferred and underwent surgery abroad at a specialized hospital. Despite 55-hour delay, the patient had a favorable outcome, returning to work 8 weeks post-surgery. This case highlights the diagnostic challenges in remote settings.

## Introduction

Acute Type A aortic dissection is a serious, life-threatening condition which often presents with severe chest and back pain accompanied by severe hemodynamic compromise. Rarely, Type A aortic dissection can present initially as critical lower limb ischemia, stroke, or myocardial infarction [1]. Mortality is high in patients with Type A aortic dissection, though timely diagnosis and subsequent surgical treatment can lower mortality [2]. Mortality can occur due to aortic valve leakage, occlusion of coronary arteries causing acute myocardial infarction, or severe bleeding in the pleural or pericardial space [2]. Diagnosis and treatment of patients with acute Type A aortic dissection in remote areas is very challenging, and timely surgery is often impossible.

## Case report

A man in his early 50s with treated hypertension presented to a remote hospital in Arctic Greenland after sudden severe chest and back pain, followed by critical pain and ischemia in his right leg. The symptoms occurred at a remote settlement and his family sailed him to hospital. After arrival, the chest and back pain had disappeared, while the patient’s right leg was still pale and cold, with no detectable pulses in the peripheral arteries, and no sensory feeling or movement in his right foot.

Initial examination showed blood pressure of 150/100 mmHg, heart rate of 50 bpm, saturation of 100%, and a temperature of 35.6°C. The ECG was normal. Blood tests showed normal levels of troponin and hemoglobin. Besides the affected leg, the general and neurological examination showed no other abnormal findings.

The local hospital only had an ultrasound scanner available, while a CT-scanner was only available at the national hospital. An acute bedside-ultrasound by a general surgeon revealed a thrombosis in the right femoral artery at the passage to arteria femoralis profunda, with cessation of Doppler flow. The ultrasound could not detect a double lumen in the thoracic or abdominal aorta. A suboptimal transthoracic FATE-scanning showed no pleural or pericardial effusion, a normal ejection fraction, and no aortic valve insufficiency.

The primary diagnosis was right femoral artery thrombosis, based on the ultrasound findings and the clinical presentation of a unilateral cold and pale leg with no detectable pulses. Thrombolysis with alteplase and enoxaparin was immediately administered at the local hospital. Since the patient had experienced severe pain from the chest and back, it was essential to investigate the causes of these symptoms further, however, due to bad weather conditions, the patient could first be transferred to the national hospital the following day.

Overnight, the right leg regained color and warmth, with pulses now detectable in both ankles, indicating that the thrombosis had dissolved.

During the morning, the patient was transferred to the national hospital, which involved a 500 km flight.

**Figure 1:**
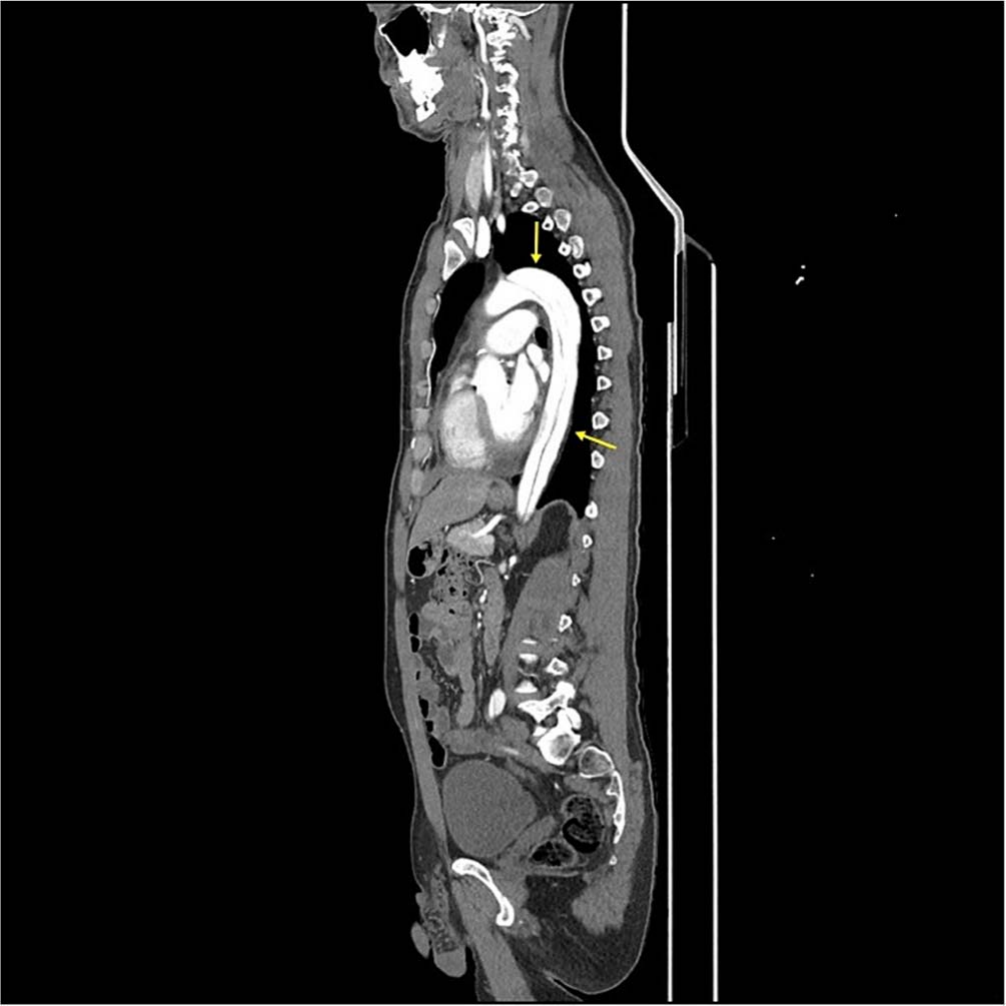
CT-scan pre-operatively showing Type A aortic dissection from the ascending aorta to the femoral arteries.

**Figure 2:**
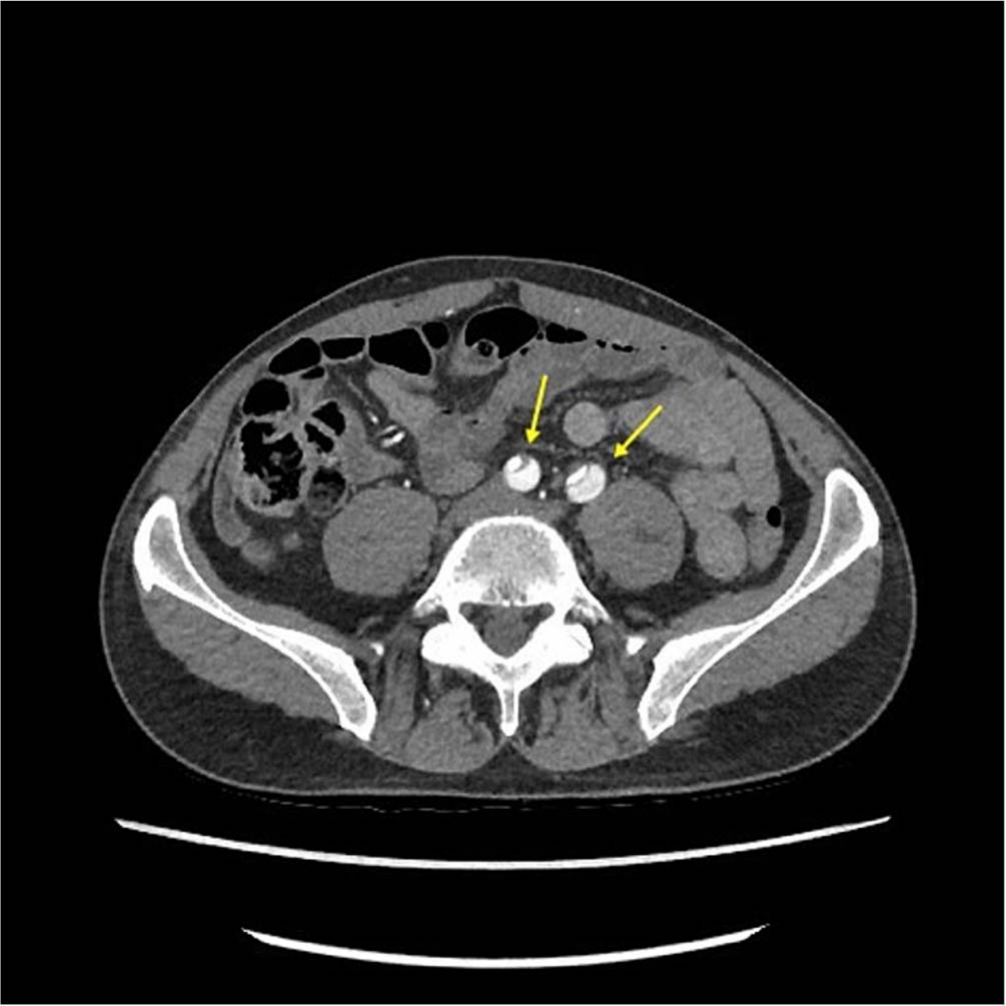
CT-scan pre-operatively showing Type A aortic dissection from the ascending aorta to the femoral arteries.

CT angiography revealed a Type A aortic dissection, stretching from the aortic valve to the femoral arteries, with cessation of blood flow halfway down the femoral artery and distally (Figs 1 and 2). An acute echocardiogram showed a dilated aortic sinus (42 mm) and ascending aorta (53 mm), with moderate aortic insufficiency. During these investigations, the patient was stable, awake, and without pain. Thrombolytic treatment was stopped immediately, and systolic blood pressure was kept <110 mmHg by labetalol and nitroglycerin.

The patient was transferred to Denmark, which involved a 3500 km flight, arriving 53 hours after initiation of symptoms. Emergency cardiothoracic surgery was performed with mechanical aortic valve replacement, and insertion of a prothesis in the ascending aorta. No bleeding complications occurred.

Three days post-surgery, he was transferred to the regular ward and discharged 9 days later. Eight weeks post-surgery he performed part-time office work, but had to retire from sled dog trips, due to persistent left leg pain and limited walking to 1000 meters. Objectively, the left foot was colder with slower capillary refill, and he experienced hyperesthesia in most of the foot.

By 12 weeks, all symptoms were dissolved. The patient was on lifelong Marevan treatment due to the mechanical valve prosthesis, with blood pressure maintained at <120/80 mmHg.

## Discussion

Diagnosing aortic dissection is challenging in remote areas. Despite symptoms of chest pain and extremity ischemia, the aortic dissection was not detected at the local hospital due to the lack of CT-scan or transesophageal ultrasound. No test has shown 100% sensitivity and specificity, so diagnosis often relies on multiple imaging methods [3].

First-line investigation when suspecting aortic dissection is often a CT-scan, as it is quick, widely available, and helps differentiate between the different types of aortic dissections. The sensitivity is reported up to nearly 100% in thoracic aortic dissections, compared to the >98% reported in MRI-scanners [4, 5].

In remote areas, hospitals often lack CT- or MRI-scanners. Ultrasound and ECG may be the only available diagnostic tools. While ECG is routine for chest pain, it is abnormal in only 30% of the patients with aortic dissection [6]. TTE has a sensitivity of 78%–90% [7], meaning that a normal ECG and TTE cannot rule out an acute aortic dissection, leaving few diagnostic tools in the acute phase to remote hospitals.

Some patients with aortic dissection experience lack of blood flow to the brain or extremities, leading to stroke or critical ischemia [8, 9], as seen in this case. In the literature, aortic dissection patients with stroke symptoms have been misdiagnosed and treated with thrombolysis [10]. While some report improved neurological outcomes [11], others have reported fatal bleeding during surgery [10, 12]. When aortic dissection is suspected but unconfirmed, both stroke and limb ischemia treatment must be carefully considered, as their symptoms overlap, but treatments differ significantly. Thrombolysis increases the risk of uncontrollable bleeding during the following surgery [13].

In our patient, thrombolysis was deliberately administered to treat the ischemic leg, resolving the ischemia 2 hours later. Given the weather circumstances preventing transfer, thrombolysis was considered safe as surgery would be delayed for many hours, in case the patient was diagnosed with aortic dissection. It is uncertain whether the right leg could have been saved without thrombolysis, as surgery was delayed due to the remote location.

This case highlights the diagnostic and therapeutic challenges of aortic dissection in remote areas.

## References

[1] Namana V, Balasubramanian R, Kariyanna PT, et al. Aortic dissection with hemopericardium and thrombosed left common iliac artery presenting as acute limb ischemia: a case report and review. Sciepub 2015;3:338–43. 10.12691/ajmcr-3-10-9.

[2] Gudbjartsson T, Ahlsson A, Geirsson A, et al. Acute type A aortic dissection – a review. Scand Cardiovasc J 2020;54:1–13. 10.1080/14017431.2019.1660401. 31542960

[3] Hines G, Dracea C, Katz DS. Diagnosis and management of acute type A aortic dissection. Cardiol Rev 2011;19:226–32. 10.1097/CRD.0b013e3182203ed9. 21808165

[4] Yoshida S, Akiba H, Tamakawa M, et al. Thoracic involvement of type A aortic dissection and intramural hematoma: diagnostic accuracy—comparison of emergency helical CT and surgical findings. Radiology 2003;228:430–5. 10.1148/radiol.2282012162. 12819341

[5] Litmanovich D, Bankier AA, Cantin L, et al. CT and MRI in diseases of the aorta. AJR Am J Roentgenol 2009;193:928–40. 10.2214/AJR.08.2166. 19770313

[6] Evangelista A, Isselbacher EM, Bossone E, et al. Insights from the international registry of acute aortic dissection: a 20-year experience of collaborative clinical research. Circulation 2018;137:1846–60. 10.1161/CIRCULATIONAHA.117.031264. 29685932

[7] Evangelista A, Flachskampf FA, Erbel R, et al. Echocardiography in aortic diseases: EAE recommendations for clinical practice. Eur J Echocardiogr 2010;11:645–58. 10.1093/ejechocard/jeq056. 20823280

[8] Gonzalez Reyes L, Perez Del Nogal G, Sierra David J, et al. Type A acute aortic dissection presenting as a stroke in a young male patient. BMJ Case Rep 2023;16:e256495. 10.1136/bcr-2023-256495. PMC10728953 38103904

[9] Khalid F, Gupta S. Aortic dissection presenting as ischemic limb. Cleve Clin J Med 2018;85:438–40. 10.3949/ccjm.85a.17122. 29883309

[10] Yin J, Meng Y, Zhang D, et al. A painful lesson: painless aortic dissection misdiagnosed as acute stroke was treated with intravenous thrombolysis. Acta Neurol Belg 2023;123:2009–11. 10.1007/s13760-022-02075-z. 36029438

[11] Kazmi SO, Achi O, Damani R. Full-dose thrombolysis for a right middle cerebral artery stroke after an acute aortic dissection. Ann Indian Acad Neurol 2018;21:223–4. 10.4103/aian.AIAN_78_18. 30258267 PMC6137638

[12] Wang J, Wu LR, Xie X. Stanford type a aortic dissection with cerebral infarction: a rare case report. BMC Neurol 2020;20:253. 10.1186/s12883-020-01832-y. 32576285 PMC7313114

[13] Matsuzono K, Suzuki M, Arai N, et al. Successful tissue plasminogen activator for a patient with stroke after Stanford type A aortic dissection treatment. J Stroke Cerebrovasc Dis 2018;27:e132–4. 10.1016/j.jstrokecerebrovasdis.2018.02.023. 29525082

